# Fe_3_O_4_@SiO_2_@VAN Nanoadsorbent Followed by GC-MS for the Determination of Polycyclic Aromatic Hydrocarbons at Ultra-Trace Levels in Environmental Water Samples

**DOI:** 10.3390/nano12172921

**Published:** 2022-08-24

**Authors:** Yu Tian, Zhigang Xu, Zhimin Liu, Xiaoxi Si, Fengmei Zhang, Wei Jiang

**Affiliations:** 1 Faculty of Science, Kunming University of Science and Technology, Kunming 650500, China; 2 Faculty of Environmental Science & Engineering, Kunming University of Science and Technology, Kunming 650500, China; 3 Yunnan Key Laboratory of Tobacco Chemistry, R&D Center of China Tobacco Yunnan Industrial Co., Ltd., Kunming 650231, China

**Keywords:** magnetic solid-phase extraction, polycyclic aromatic hydrocarbons, vancomycin, GC-MS

## Abstract

In the present study, silica-coated magnetic nanoparticles functionalized with vancomycin (Fe_3_O_4_@SiO_2_@VAN) were synthesized. The Fe_3_O_4_@SiO_2_@VAN nanocomposite was used as a sorbent for the magnetic solid-phase extraction (MSPE) of polycyclic aromatic hydrocarbons (PAHs) from environmental water, followed by GC-MS. The nanocomposite was characterized by Fourier-transform infrared spectroscopy, X-ray diffraction, scanning electron microscopy, transmission electron microscopy, vibrating sample magnetometry, and nitrogen sorption. Various experimental parameters were optimized, including extraction condition and desorption condition. Results show that Fe_3_O_4_@SiO_2_@VAN combined the advantages of nanomaterials and magnetic separation technology, showing excellent dispersibility and high selectivity for PAHs in environmental water sample. Under the optimal extraction conditions, an analytical method was established with the sensitive limit of detection (LOD) of 0.03–0.16 μg L^−1^. The method was successfully applied for the analysis of environmental water samples. The relative standard deviations (%) were in the range of 0.50–12.82%, and the extraction recovery (%) was in the range of 82.48% and 116.32%. MSPE-coupled gas chromatography–mass spectrometry quantification of PAHs is an accurate and repeatable method for the monitoring of PAH accumulation in environmental water samples. It also provides an effective strategy for the tracing and quantification of other environmental pollutants in complex samples.

## 1. Introduction

Polycyclic aromatic hydrocarbons (PAHs) are a class of cyclic organic compounds consisting of two or more dense aromatic rings, forming a large and diverse group of organic compounds [1]. PAHs exhibit carcinogenic and teratogenic activities, and their carcinogenicity increases with the number of benzene rings [2,3]. The low water solubility and high octanol–water partition coefficient PAHs present lead to low bioavailability and high levels of accumulation in the environment, resulting in environmentally persistent, bioconcentrated, and difficult to degrade PAHs [4,5]. The European Union Water Framework Directive, the United States Environmental Protection Agency (USEPA), and the European Union (EU) have placed 16 PAHs on the list of priority persistent organic pollutants for control [6,7,8]. The US EPA and the Chinese national environmental quality standards have set recommended PAH concentrations of 0.2 and 2.0 μg L^−1^ for drinking water, respectively (USEPA 1993, GB5749-2006). PAHs widely exist in the environment, and this condition can lead to water and food pollution; these PAHs can also enter the human body by food chains [9]. Therefore, the sensitivity of the detection methods used for PAHs needs to be improved.

Currently, several analytical techniques have been reported for PAH detection, including high-performance liquid chromatography (HPLC) [10], gas chromatography (GC) [11,12,13], and gas chromatography–mass spectrometry (GC-MS) [14,15]. GC-MS is the most common method used for PAH detection in wastewater because of its rapid implementation and high detection sensitivity. However, the trace amounts in the environment, as well as hydrophobic and matrix interference in actual samples, make PAHs difficult to detect directly. Therefore, the sensitivity of the detection methods used for PAHs, especially for multi-PAHs in environmental water, needs to be improved. Some modern sample pre-treatment methods are used for the analysis of PAHs from complex matrix, including QuEChERS [16], accelerated solvent extraction (ASE) [17], molecular imprinting methods (MIPs) [18], solid-phase extraction (SPE), and magnetic solid-phase extraction (MSPE) [19,20]. MSPE is a simple and green pretreatment technology which is based on magnetic nanoparticles (MNPs) adsorbents consisting of Fe_3_O_4_ nanoparticles coated with special functional groups. However, Fe_3_O_4_ and other MNPs are prone to agglomeration in aqueous solution, which could affect their particle size, surface characteristics, and adsorption performance [21]. To address these mentioned difficulties, bare MNPs can be protected through surface modification and functionalization [22].

The main challenge in the extraction of PAHs from environmental water is the lack of the functional groups in their structures, as they cannot be targeted for extraction. In most cases, the matrix is aqueous or hydrophilic, and the analyte is hydrophobic, resulting in low compatibility of the sorbent with the matrix and thus limiting the effectiveness of the analysis method. Vancomycin (VAN), a glycopeptide macrocyclic antibiotic, is well known as a host molecule. It has a complex and unique molecular structure that can adsorb analytes in various ways, including hydrophobic interactions, electrostatic interactions, hydrogen bonding, dipole superposition, and π-π conjugation [23], thus providing an opportunity to accommodate different guest molecules. Therefore, vancomycin shows great promise for applications such as host–guest recognition and adsorption separation. Considering the large π-plane hydrophobicity polycyclic aromatic hydrocarbons, the hydrophobic effect can be used to warp water-insoluble organic guest molecules into the cavity of vancomycin. Furthermore, vancomycin has a unique porous space and shows good adsorption properties to polycyclic aromatic hydrocarbons. The PAHs in the cavity are stable by various interactions of many atoms and functional groups. The most important interactions are π-π interactions and hydrophobic interactions. In addition to these types of interactions, inclusion complexation, dipole interactions, van der Waals forces and spatial site resistance effects also may play a crucial role in adsorption stabilization. In this way, these PAH molecules are stable in the vancomycin cavity and the PAHs are then eluted from the cavity by the selection of a suitable eluent. Then, organic VAN is deposited on the surface of Fe_3_O_4_@SiO_2_ MNPs to improve the surface area and adsorption properties. Hence, VAN can be used as an adsorbent immobilized on magnetic composites for the purification and separation of analytes, effectively combining the high adsorption capacity of VAN with the ease of separation of magnetic nanomaterials.

In the present work, we present a high-sensitivity, anti-matrix interference, simple, and reliable MSPE method based on magnetic adsorbent (Fe_3_O_4_@SiO_2_@VAN) for the preconcentration of PAHs in real samples. Desorption isotherms can be classified as having a high selectivity for target PAHs. Under the optimized extraction conditions, the proposed method was applied for the detection of PAHs in environmental water.

## 2. Materials and Methods

### 2.1. Chemicals and Materials

Phenanthrene (PHE, 99%), Anthracene (AN, 99%), Fluoranthene (FA, 99%), Pyrene (Py, 99%), Benzo(a)anthracene (BaA, 99%), and Benzo(b)fluoranthene (BbFA, 99%) were purchased from Aladdin Chemistry Co., Ltd. (Shanghai, China). Vancomycin (VAN, >900 μg mg^−1^) was obtained from Shanghai Civi Chemical Technology Co., Ltd. (Shanghai, China). Ethylene glycol (99.8%), FeCl_3_·6H_2_O (99%), polyethylene glycol (PEG-2000), tetraethyl orthosilicate (TEOS, 98%), γ-glycidoxypropyltrimethoxysilane (KH560), and sodium acetate (NaAc, 99%) were supplied from Aladdin (Shanghai, China). Acetonitrile, methanol, anhydrous ethanol (EtOH), and *N*,*N*-dimethylamide (DMF, 99.8%) were acquired from Merck Co., Ltd. (Shanghai, China). Distilled water was purchased from A.S. Watson Group Co., Ltd. (Fujian, China). Hydrochloric acid (37%) was bought from Aladdin (Shanghai, China).

### 2.2. Instrumentation

Chromatographic separation and identification of PAHs were performed by GC-MS technology (Clarus 600, Salt Lake City, UT, USA). A DB-5 fused-silica column (30 m × 0.25 mm, 0.25 μm film thickness; Agilent J&W, Santa Clara, CA, USA) was used for chromatographic separation and identification of PAHs. The morphologies of magnetic nanomaterials were characterized by using a Nova Nano SEM450 scanning electron microscope (SEM, FEI, Hillsboro, OR, USA) and a Tecnai G2 TF30 transmission electron microscope (TEM, FEI, Hillsboro, OR, USA). The phase and crystalline structures of magnetic adsorbent were measured with an X-ray diffractometer (XRD, PANalytical Empyrean, Almelo, The Netherlands) and a Tensor 27 FT-IR spectrometer (Bruker, Bremen, Germany). The surface area and porosity analysis of magnetic nanomaterials were carried out with a nitrogen adsorption instrument (AUTO SORBIQ-MP, Quantachrome, Boynton Beach, FL, USA). The saturation magnetization of fabricated magnetic nanocomposites was measured on a vibra sample magnetometer (VSM) model Versa Lab from Quantum Design (USA). The 0.22 μm organic phase membrane was bought from Tianjin jinteng Experimental Equipment Co., Ltd. (Tianjin, China).

### 2.3. Synthesis of Fe_3_O_4_@SiO_2_@VAN Nanoparticles

#### 2.3.1. Preparation of the Fe_3_O_4_ Nanoparticles

A solvothermal method was employed to prepare Fe_3_O_4_ nanoparticles. In a typical synthesis procedure, FeCl_3_·6H_2_O (2.7 g) was dissolved in ethylene glycol (50 mL) and mechanically stirred vigorously for 30 min. Subsequently, NaAc (7.2 g) and PEG-2000 (2.0 g) were added in the above mixture and stirred well. Then, the homogenized mixture was transferred into the Teflon lined stainless-steel autoclave and then heated, at 200 °C, for 8 h. The Fe_3_O_4_ nanoparticles were washed for several times with EtOH and water until reaching neutrality, and then dried, at 60 °C, for 24 h.

#### 2.3.2. Preparation of Core–Shell Fe_3_O_4_@SiO_2_ Nanoparticles

The Fe_3_O_4_ nanoparticles were coated silica shells by sol–gel co-condensation. In a typical reaction, Fe_3_O_4_ (1.0 g) was dispersed in 1 mol L^−1^ of HCl by ultrasonic for 10 min. Afterward, deionized water (20 mL) and ammonia (2.5 mL) were added. The mixture was agitated for 30 min under vigorous mechanical stirring. Subsequently, 0.5 mL of TEOS was added into the above mixture, and then continuously stirred for 12 h. After the reaction, the Fe_3_O_4_@SiO_2_ magnetic particles were obtained. It was washed with ethanol and water repeatedly and finally dried, at 60 °C, for 24 h. Fe_3_O_4_@SiO_2_ nanoparticles were eventually obtained.

#### 2.3.3. Synthesis of Fe_3_O_4_@SiO_2_@VAN Nanoparticles

**Scheme 1:**
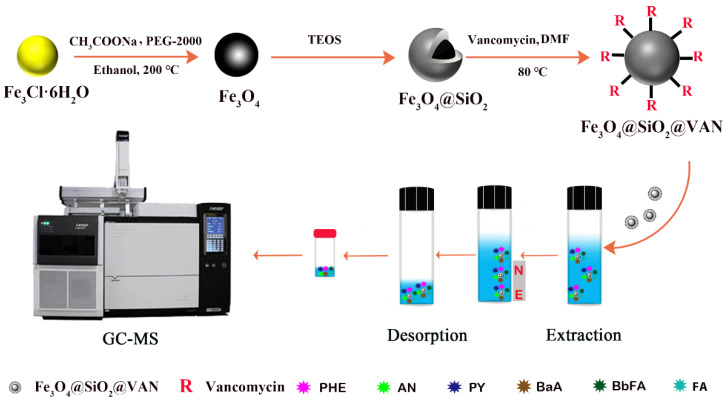
Schematic procedure for Fe_3_O_4_@SiO_2_@VAN magnetic composite particles.

Firstly, vancomycin (VAN, 13.82 g) and γ-glycidoxypropyltrimethoxysilane (1.88 mL) were dissolved in DMF (150 mL), and stirred, at 80 °C, for 24 h. Subsequently, Fe_3_O_4_@SiO_2_ (2.5 g) was added to the above mixture then stirred, at 80 °C, for 24 h. Afterward, the obtained Fe_3_O_4_@SiO_2_@VAN nanoparticles were washed for several times with water and ethanol. The resulting Fe_3_O_4_@SiO_2_@VAN nanoparticles were dried, at 60 °C, for 24 h. The procedure for the fabrication of Fe_3_O_4_@SiO_2_@VAN magnetic adsorption material is shown in Scheme 1.

### 2.4. Extraction Procedure

In total, 50 mg Fe_3_O_4_@SiO_2_@VAN nanoparticles was added to 30 mL of 100 µg L^−1^ PAH solution. The mixture was stirred in a vortex with a stirrer at 250 rpm for 1 h to favor the extraction of the target analytes. Then, the sorbent was isolated from the solution by using a magnet, and then transferred into a 1.5 mL glass vial with 1 mL of n-hexane. The sorbent was sonicated for 10 min, and then filtered through a 0.22 μm organic phase membrane. The desorption solution was collected in a 1.5 mL glass vial for further GC-MS analysis.

### 2.5. Instrument Conditions

Chromatographic separation and identification of PAHs were carried out on a GC–MS system (Clarus 600, USA) by using a capillary column DB-5 (30 m × 0.25 mm, 0.25 μm film thickness, Agilent J&W). The carrier gas was helium at a flow rate of 1.2 mL min^−1^. The injector and ion source temperatures were set at 250 and 260 °C, respectively. The initial temperature of column was set at 80 °C for 2 min, further raised to 180 °C at 20 °C min^−1^ and held for 2 min, and then raised to 230 °C at 3 °C min^−1^ and held for 2 min. Finally, the temperature was increased to 300 °C at 30 °C min^−1^, and then held for 8 min. The temperature of the GC inlet was 250 °C. The solvent delay time was 5 min. The transmission line temperature was 280 °C. Mass spectrometry detection was carried out in the selected ion detection mode.

## 3. Results and Discussion

### 3.1. Characterization of Materials

**Figure 1:**
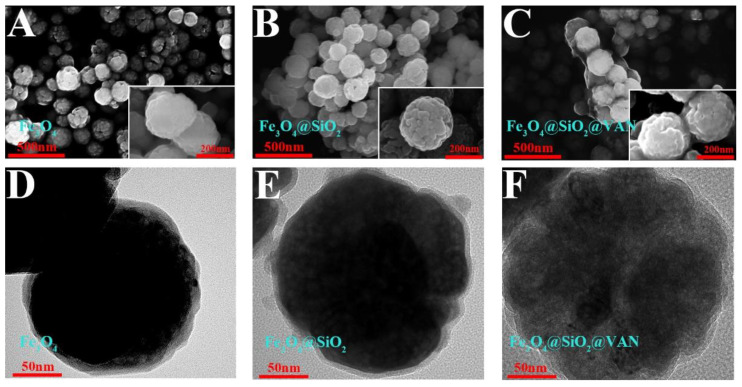
SEM and TEM images of three nanoparticles. (**A**,**D**) Fe_3_O_4_ nanoparticles; (**B**,**E**) Fe_3_O_4_@SiO_2_ nanoparticles; (**C**,**F**) Fe_3_O_4_@SiO_2_@VAN nanoparticles.

The surface morphology and microstructure of Fe_3_O_4_, Fe_3_O_4_@SiO_2_, and Fe_3_O_4_@SiO_2_@VAN nanoparticles were observed using SEM and TEM. The SEM images of Fe_3_O_4_, Fe_3_O_4_@SiO_2_, and Fe_3_O_4_@SiO_2_@VAN nanoparticles are displayed in Figure 1A–C. It can be seen that Fe_3_O_4_ nanoparticles are uniform in size, spherical in shape, and approximately 200 nm in diameter. After being coated with a thin, transparent cladding, a silica layer, a typical morphology with core/shell structure, was obtained. The dark Fe_3_O_4_@SiO_2_@VAN nanoparticles were obtained after being coated with VAN on the surface of Fe_3_O_4_@SiO_2_, and the diameter of Fe_3_O_4_@SiO_2_@VAN roughly 210–230 nm. The TEM images of Fe_3_O_4_, Fe_3_O_4_@SiO_2_, and Fe_3_O_4_@SiO_2_@VAN nanoparticles are shown in Figure 1D–F. The figure shows that the dark Fe_3_O_4_ surfaces are covered by transparent silica layer alone, forming a uniform amorphous shell that can protect the core–shell of the Fe_3_O_4_ magnetic nanoparticles and makes further modification more effective. The thin and transparent shell, approximately 10 nm thick, is the silica layer. After Fe_3_O_4_@SiO_2_ composites were wrapped by VAN, the surfaces of the microspheres became rough. Therefore, the magnetic nanosorbent facilitates the adsorption of the target analyte, thus increasing the adsorption ability.

The FT-IR results are shown in Figure S1. The peaks at 578 and 1623 cm^−1^ are related to the Fe–O and hydroxyl groups on the surface of Fe_3_O_4_ nanoparticles, respectively (spectrum (a)). The absorption peaks at 462, 796, and 1101 cm^−1^ were observed in spectrum (b), along with corresponding bending vibration mode Si-O-Si and the presence of Si–O–Si symmetrical and asymmetrical stretching vibration [24,25]. In addition, the peak occurring around 956 cm^−1^ expressed the stretching vibration absorption of Fe–O–Si, confirming that the Fe_3_O_4_@SiO_2_ has been successfully prepared [26]. The Fe_3_O_4_@SiO_2_@VAN FT-IR spectrum (spectrum (c)), which formed after the Fe_3_O_4_@SiO_2_ NPs were coated with VAN, exhibited an absorption peak at 1230 cm^−1^ relates to benzene that belong to VAN molecule [27]. In addition, the peak of the stretching vibration peaks at 3280, 1660, and 1047 cm^−1^ correspond to vancomycin, indicating that VAN successfully bonded with Fe_3_O_4_@SiO_2_ [28].

**Figure 2:**
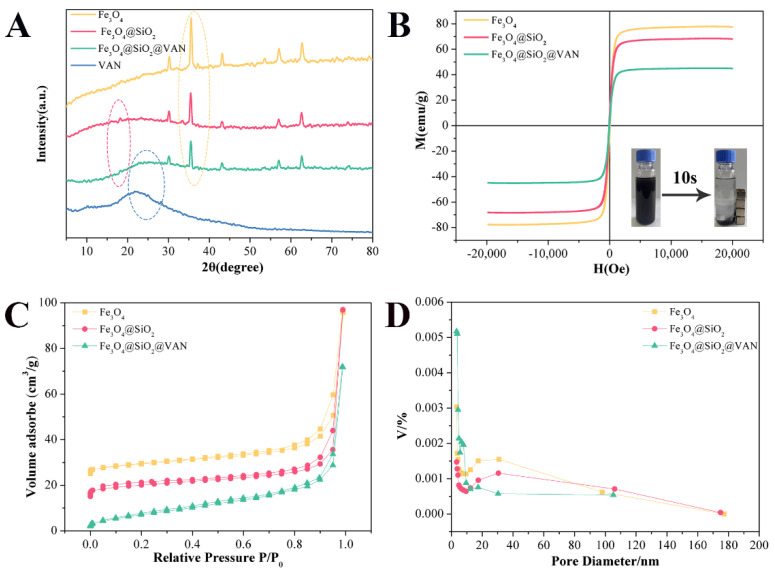
(**A**) XRD spectra; (**B**) VSM magnetization curves; (**C**) Nitrogen adsorption–desorption isotherms; (**D**) Pore diameter distribution.

The phases and crystalline structures of Fe_3_O_4_, Fe_3_O_4_@SiO_2_, and Fe_3_O_4_@SiO_2_@VAN nanocomposites were confirmed by XRD nanocomposites. As illustrated in Figure 2A, the formation of Fe_3_O_4_ material was confirmed based on the peaks of 2θ at 30.28°, 35.60°, 43.26°, 53.73°, 57.28°, and 62.81°, which were assigned to the (220), (311), (400), (422), (511), and (440) of standard data of Fe_3_O_4_ diffraction peak [29,30,31], respectively. After silica shell loading with Fe_3_O_4_ nanocomposites, peak alterations were observed with the reduction in the peak intensity of Fe_3_O_4_@SiO_2_. Therefore, the silica shell did not change the crystal structure of Fe_3_O_4_. Moreover, a broad peak can be seen in the 2θ of 19–27° in Fe_3_O_4_@SiO_2_@VAN nanoparticles pattern, which was related to VAN, indicating that the Fe_3_O_4_@SiO_2_ surface was successfully coated with VAN.

It is critical that the magnetic nanomaterials should possess magnetic property for their rapid separation in removing process. Therefore, the magnetization curves of nanomaterials were obtained using vibrating sample magnetometer (VSM), as illustrated in Figure 2B. The saturation magnetization values (Ms) of Fe_3_O_4_, Fe_3_O_4_@SiO_2_, and Fe_3_O_4_@SiO_2_@VAN nanoparticles were 77.6, 68.3, and 44.18 emu g^−1^, respectively, indicating that the magnetic response of nanomaterials reduced gradually with further modification, directly confirming the functional materials are modified on magnetic Fe_3_O_4_ individually. In addition, all of these three kinds of samples show the fast magnetic response, which provides the samples with ability of magnetic separation and collection. The Fe_3_O_4_@SiO_2_@VAN nanocomposite material could be dispersed evenly in the solution matrix and quickly separated from aqueous phase by a magnet within 10 s (inset in Figure 2B).

The porous structure, pore distribution and specific surface area of as-prepared three nanomaterials were investigated using BET method. As shown in Figure 2C,D, the nitrogen adsorption–desorption isotherms can be classified as IV curves, confirming the existence of mesoporous structure. Compared with Fe_3_O_4_@SiO_2_, the ratio of mesopores on the surface of Fe_3_O_4_@SiO_2_@VAN nanomaterials noticeably increased, which could offer more potential interaction sites and spatial match effects for the extraction of PAHs with high steric hindrance. Table S1 summarizes some textural parameters. It shows that the pore volumes of Fe_3_O_4_@SiO_2_@VAN (0.108 cc g^−1^) are less than that of uncoated Fe_3_O_4_ nanomaterials 0.128 (cc g^−1^), which is due to the pore blockage of the Fe_3_O_4_@SiO_2_@VAN surface.

### 3.2. Optimization of Extraction Conditions

Before the analytical method validation, the key parameters of extraction condition (time, salt addition and pH of the sample) and desorption condition (solvent, time) were optimized to obtain the best extraction efficiency. The initial experimental conditions were extraction aqueous solution concentration of 0.1 mg L^−1^. The extraction aqueous solution and the desorbing solution volumes were 30 and 1.0 mL, respectively. The extraction and desorbing time were 30 and 10 min, respectively. All experiments were tested six times in parallel. The extraction amount (*Q*, μg) of analyte was calculated using Equation (1). The extraction recovery was calculated using Equation (2).

(1) $${Q=V\;(C}_{0}-C_{e})\;$$

(2) $$\mathrm{Extraction}\;\mathrm{recovery}=\frac{Q}{Q_{0}}\;\times \;100$$

where *C*_0_ (mg L^−1^) is the initial concentration of PAHs before extraction, *C_e_* (mg L^−1^) is the equilibrium concentration of extraction solution of PAHs after by Fe_3_O_4_@SiO_2_@VAN, V (mL) is the volume of the extraction solution of PAHs, *Q*_0_ (μg) is the initial quantity of PAHs, and *Q* (μg) is the equilibrium binding quantity of PAHs on the Fe_3_O_4_@SiO_2_@VAN.

#### 3.2.1. Extraction and Desorption Time Optimization

Extraction time should be sufficient to achieve the extraction equilibrium. Thus, the effect of extraction time for six PAHs was studied in the range of 2–120 min. It can be seen that the extraction efficiency increased in the first 30 min and basically stabilized afterward (Figure S2A). This phenomenon is caused by the saturation of analytes on the sorbent sites over time. The desorption time profile on extraction performance was attained at a time duration of 2–20 min (Figure S2B). The highest recovery obtained when the desorption time was 5 min.

#### 3.2.2. Desorption Solvent, pH, and Salt Addition Optimization

To select the best desorption solvent, we investigated different eluents such as dichloromethane, n-hexane, ethanol, and acetonitrile and evaluated their extraction efficiencies. According to the results in Figure S2C, dichloromethane exhibited the best desorption performance. Therefore, dichloromethane was chosen as the best desorption solvent. In water solution, pH is an essential factor for the extraction performance by affecting the stability of the sorbent, the charge on its surface and analytes. In order to obtain the optimum pH, we studied various pH values in the range of 2.0–12.0. Since the PAHs are neutral compounds, the pH of the sample may not have a significant effect on the extraction efficiency of PAHs, but pH can affect the morphology and dispersion of the magnetic materials in the aqueous phase. Acidic and basic conditions may affect the dispersion of the sorbent in water and some leaching of Fe_3_O_4_@SiO_2_@VAN magnetic material. The best extraction efficiency was obtained at pH 7.0, and this phenomenon is due to the zwitterionic form of PAHs, which has a strong binding affinity with the cavity of VAN (Figure S2D). In the ionic strength range of 0–25%, The influence of ionic strength was examined (Figure S2E). The presence of NaCl would decrease the solubility of the analytes by salting-out effect and would thus be conducive to the extraction of PAHs by Fe_3_O_4_@SiO_2_@VAN. However, after the salt concentration exceeded 20%, analyte recoveries were reduced in viscosity of the solution, thus hindering the mass transfer of analytes. Therefore, 20% NaCl was added to the extraction solution.

### 3.3. Selectivity Evaluation

Under the optimal conditions of 100 μg L^−1^ concentration of extraction solution, 30 mL volume, 30 min extraction time, 1 mL volume of desorbing solution in dichloromethane, and 20% NaCl addition to the extraction solution, five substances were selected for the selectivity study of the non-structural analogues, such as bisphenol A, bisphenol B, pyridine, and phenol, and structural analogues (one of the polycyclic aromatic hydrocarbons) such as acenaphthylene. The enrichment factor (*EF*) and selectivity coefficient (*SC*) were calculated using Equations (3) and (4) as follows:

(3) $$\mathrm{EF}=\frac{C_{f}}{C_{i}}\;$$

(4) $$\mathrm{SC}=\frac{{\mathrm{EF}}_{p}}{{\mathrm{EF}}_{c}}$$

where *C_i_* (μg L^−1^) is the initial concentration before extraction, and *C_f_* (μg L^−1^) is the concentration of analytes after Fe_3_O_4_@SiO_2_@VAN extraction. *EF_p_* and *EF_c_* are the enrichment factors for PAHs and its competitors.

**Figure 3:**
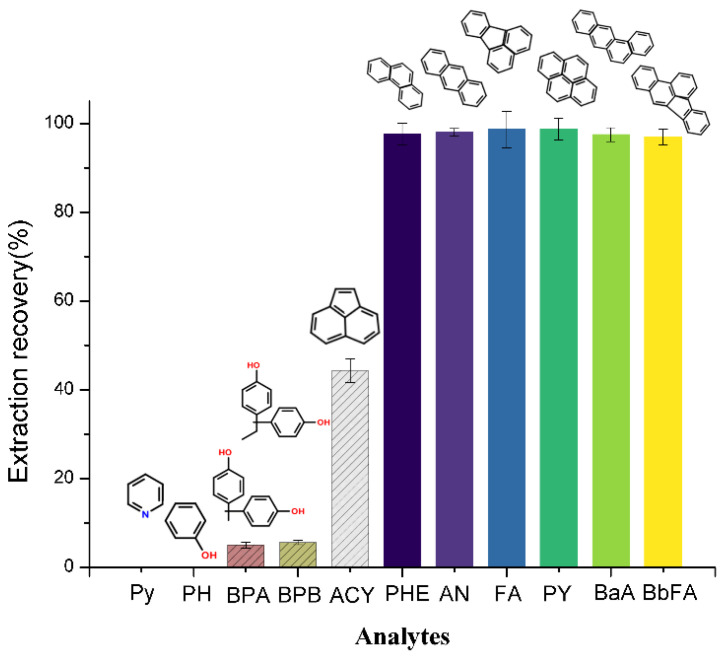
The selectivity evaluation of Fe_3_O_4_@SiO_2_@VAN nanomaterials.

As shown in Figure 3, the magnetic sorbent material has the best extraction effect for the target analytes (weak polarity and consisting of dense aromatic rings), which all exceeded 90%, whereas no extraction effect was observed for pyridine and phenol, which are more polar and consist of one aromatic ring. For the homologue acenaphthene, due to its higher structural similarity, the vancomycin magnetic material (Fe_3_O_4_@SiO_2_@VAN) had a better extraction effect on it with an extraction recovery of 45%, but considering its shorter benzene ring chain, the extraction effect was significantly lower than that of the six target analytes. Meanwhile, we list the solubility coefficient (S_w_) and octanol–water partition coefficient (log*K_ow_*) of PAHs in Table S2. As the number of thickened benzene rings increases, the S_w_ decreases and hydrophobicity of PAHs increases, which can cause the Fe_3_O_4_@SiO_2_@VAN material to have an excellent adsorption capacity for aromatics with multi-benzene rings due to hydrophobic interactions. Therefore, the sorbent has a good selectivity for PAHs, which can be attributed to the unique multi-ring cavity structure and hydrophobic interaction between them. For the determination of the selectivity of Fe_3_O_4_@SiO_2_@VAN, Table S3 exhibits the calculated extraction recovery, EF and SC values. The extraction recovery of PAHs was 97.0–98.0%, and the corresponding EF values were 51.2, 58.0, 53.0, 52.6, 54.0, and 50.0 for PHE, AN, FA, PY, BaA, and BbFA, respectively. The SC values were in the range of 5.0–34.1, suggesting that Fe_3_O_4_@SiO_2_@VAN possesses high adsorption selectivity for PAHs.

### 3.4. Establishment of Analytical Methods

To further explore the application of Fe_3_O_4_@SiO_2_@VAN for the detection of PAHs in real samples, a matrix matching standard analytical method was established. The linear range was 0.1–200 μg L^−1^. The linear equations and correlation coefficients are shown in Table 1. When the signal-to-noise ratio (S/N) is 3 and 10, respectively, the limit of detection (LOD) and limit of quantification (LOQ) of PAHs are obtained. The resulting LODs were in the range of 0.03–0.16 μg L^−1^, and LOQs were in the range of 0.090–0.48 μg L^−1^. The US EPA and Chinese national environmental quality standards have set the recommended PAH concentrations to 0.2 and 2.0 μg L^−1^ for drinking water, respectively (USEPA 1993, GB5749-2006), which are higher than the highest LOD (0.16 μg L^−1^) obtained from our proposed method. Therefore, the proposed method can meet the detection of allowable PAHs content in water samples.

### 3.5. Detection of Actual Samples

**Figure 4:**
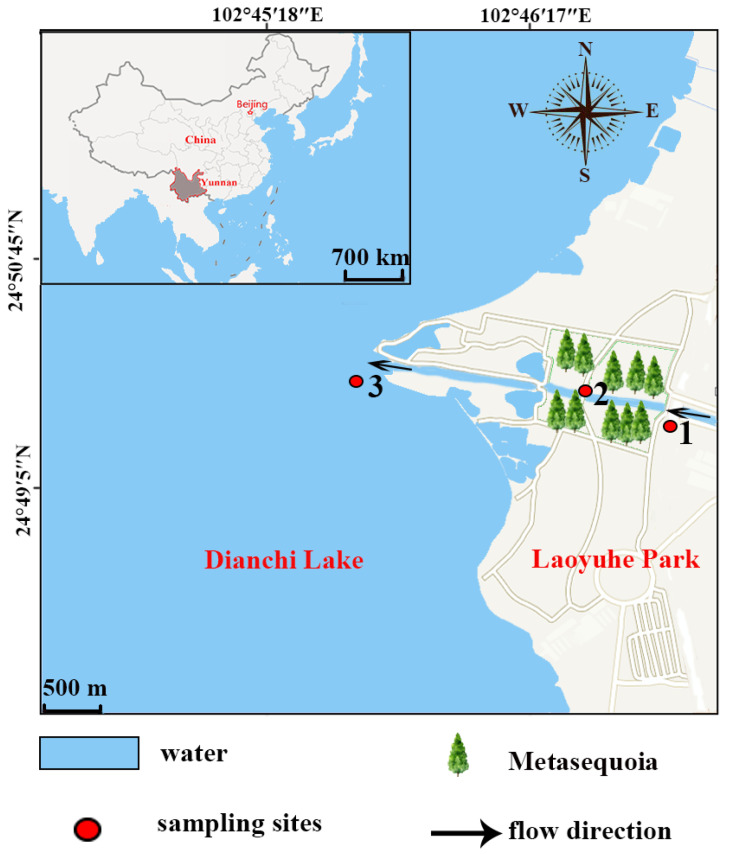
Geographical distribution of sampling sites.

Over the past several decades, the rapid population growth and economic development in the Dianchi Lake basin have resulted in the discharge of many contaminants into the lake, and PAHs have become the main pollutant. Accordingly, the Kunming government has spent enormous human and financial resources to rectify the Dianchi Lake by building Laoyuhe constructed wetlands. Plants play an important role in the biogeochemical cycle of environmental pollutants. PAHs in the rhizosphere soil have strong bioaccumulation potential for root absorption. Therefore, *Metasequoia glyptostroboides* is used for the treatment of polluted Dianchi Lake because of its strong root system. The overall structure of the artificial wetland in the Laoyuhe River is shown in Figure 4, Urban wastewater flows from point 1 into the Luyu River Park, is degraded by *M. glyptostroboides* when it flows through point 2, and then flows into Dianchi at point 3.

To evaluate the above analytical method for genuine samples by using Fe_3_O_4_@SiO_2_@VAN as sorbents for MSPE in combination with GC-MS for detection, we obtained environmental water samples from three different points of the Laoyuhe River in Kunming City (Figure 4).

To eliminate possible matrix effects, a standard addition method was adopted for the quantitative determination of PAHs. Three aliquots of the lake sample were analyzed in parallel. The results showed that only pyrene was found in three sampling points with different concentrations. The concentrations of pyrene from the inlet to the outlet are 0.35, 0.26, and 0.14 μg L^−1^, respectively. The gradual decrease in PAH concentration from the inlet to the outlet in Laoyuhe River suggests that *M. glyptostroboides* is an effective pathway for perennial tree species for the removal of PAHs absorbed into them, and the Yunnan government has achieved significant and positive results in the management of Dianchi Lake. Furthermore, the analysis of PAHs in environmental water samples has a good recovery, with the range of 82.48–116.32%, and the RSD% (*n* = 5) does not exceed 12.82% (Table 2). The chromatogram of sample is displayed in Figure S3.

### 3.6. Comparison with Other Reported Methods

The developed method by using Fe_3_O_4_@SiO_2_@VAN as MSPE adsorbent material was compared with previously reported methods (Table 3). Results show that the developed approach for PAHs analysis was more sensitive than those in previously reported methods. The proposed method exhibited low LODs, eco-friendliness, and anti-matrix interference for monitoring of target PAHs. However, the new approach showed the longest extraction time values. This fact can be ascribed to the use of extraction method. Although sonication can accelerate the adsorption of analytes to the material, it may also cause irreversible damage to the material. Therefore, we choose a gentle method (stirring) for the adsorption of analytes to the material.

## 4. Conclusions

In this research, we proposed an easy-to-operate, high sensitivity, and anti-matrix interference MSPE method based on a functionalized magnetic nanoadsorbent (Fe_3_O_4_@SiO_2_@VAN) followed by GC-MS for the preconcentration and detection of PAHs in natural water samples. The synthesized novel magnetic material exhibit nano- and mesoporous structures, as well as superparamagnetic and excellent hydrophobic properties. High selectivity for the target analytes and good dispersibility in hydrophilic matrices were significant advantages of the nanoadsorbent. Under the most favorable extraction conditions, wide linear range, low LODs, good RSDs, and high relative recovery were obtained. Finally, this method was applied for determining PAHs in environmental water samples, and satisfactory results were achieved. These remarkable features provide the great potential of Fe_3_O_4_@SiO_2_@VAN for the extraction of other hydrophobic analytes from water samples.

## Figures and Tables

**Table 1:** Linear equations, detection limit and limit of quantification of six kinds of PAHs.

Analyte	LODs (μg L^−1^)	LOQs (μg L^−1^)	Linear Range (μg L^−1^)	Linear Equation	Correlation Coefficient (R)
phenanthrene	0.03	0.09	0.1–200	Y = 862X-701	0.9923
anthracene	0.03	0.09	0.1–200	Y = 1302X-1121	0.9863
fluoranthene	0.03	0.09	0.1–200	Y = 1612X-1190	0.9997
pyrene	0.03	0.09	0.1–200	Y = 4707X-2273	0.9906
benzo[a]fluoranthene	0.03	0.09	0.1–200	Y = 1956X-1525	0.9945
benzo[b]fluoranthene	0.16	0.48	0.5–200	Y = 1883X-1431	0.9963

**Table 2:** The established method and method validation of PAHs in water samples.

Sites	Analyte	Found (μg L^−1^)	Spiked Samples Analysis					
			1 μg L^−1^		50 μg L^−1^		100 μg L^−1^	
			Recovery (%)	RSD (%)	Recovery (%)	RSD (%)	Recovery (%)	RSD (%)
Site 1	phenanthrene	/	95.70	1.13	116.32	1.21	96.96	2.73
	anthracene	/	92.31	2.60	112.00	1.55	106.07	0.89
	fluoranthene	/	99.60	11.90	110.80	2.07	91.30	2.31
	pyrene	0.35	95.63	8.25	95.68	4.32	99.56	4.36
	Benzo[a]anthracene	/	97.64	5.26	119.00	3.71	98.23	3.85
	Benzo[b]fluoranthene	/	101.91	6.23	101.10	5.67	88.22	0.50
Site 2	phenanthrene	/	95.00	1.94	98.76	2.60	96.82	6.41
	anthracene	/	85.63	12.82	89.00	5.04	105.11	3.94
	fluoranthene	/	100.00	2.02	86.62	4.15	103.47	4.78
	pyrene	0.26	98.18	2.80	85.40	6.31	107.00	10.22
	Benzo[a]anthracene	/	111.40	6.59	82.48	11.14	102.12	3.36
	Benzo[b]fluoranthene	/	87.32	8.08	91.87	11.72	102.47	2.79
Site 3	phenanthrene	/	82.49	3.06	97.00	4.24	102.00	2.56
	anthracene	/	74.52	0.14	93.36	4.20	102.38	3.05
	fluoranthene	/	77.53	4.26	101.26	3.57	99.73	5.74
	pyrene	0.14	100.00	3.69	103.56	2.30	101.94	3.16
	Benzo[a]anthracene	/	85.63	9.08	94.04	1.01	101.49	0.52
	Benzo[b]fluoranthene	/	99.32	8.42	93.29	2.91	99.30	6.51

“/” means PAHs not found.

**Table 3:** Comparison of the published methods for PAHs analysis with the method proposed in this work.

Sample	Sample Preparation	Sorbent	Adsorption Amount (mg)	Extraction Time (min)	Limit of Detection (μg L^−1^)	Detection Methods	References
water	MSPE	OPA/MMNPs	50	1 (vortexed)	0.016–0.07	GC-MS	[32]
water	MSPE	Magnetic MIL-100(Fe)	10	10 (ultrasounds)	0.032–2.11	HPLC-FLD	[33]
water	MSPE	g-C_3_N_4_/Fe_3_O_4_	10	10 (ultrasounds)	0.05–0.10	HPLC-FLD	[34]
water	MSPE	Fe_3_O_4_/GO	40	10 (shaking)	0.09–0.19	HPLC-UV	[35]
water	MSPE	Fe_3_O_4_@COF–COOH	10	10 (ultrasounds)	0.025–10.0	HPLC-DAD	[36]
water	MSPE	Carbon-ferromagnetic nanocomposite	10	30 (shaking)	0.015–0.335	GC-MS	[37]
seawater	MSPE	PI@SiO_2_@Fe_3_O_4_	50	20 (stirring)	0.15–0.19	GC-FID	[38]
tap, river, well and reservoir waters	MSPE	Fe_3_O_4_@SiO_2_@MIM-PF6	30	7 (ultrasounds and stirring)	0.04–1.11	GC-MS	[39]
tap water	MSPE	Magnetic C18 microspheres	50	1 (ultrasounds)	0.8–36	GC-MS	[40]
water	MSPE	Fe_3_O_4_@SiO_2_@VAN	50	30 (stirring)	0.03–0.16	GC-MS	This work

## Data Availability

Not applicable.

## Supplementary Materials

The following supporting information can be downloaded at: https://www.mdpi.com/article/10.3390/nano12172921/s1, Figure S1: FT-IR spectra of (a) Fe_3_O_4_, (b) Fe_3_O_4_@SiO_2_, (c) Fe_3_O_4_@SiO_2_@VAN and (d) VAN; Figure S2: (A) Extraction time optimization; (B) Desorption time optimization; (C) Desorption solvent optimization; (D) Solution pH optimization; (E) The effect of solution ionic strength on the extraction effect of three kinds of PAHs; Figure S3: Selected ion chromatogram of Laoyuhe River water sample. (a) extraction chromatographic analysis after adding 1 μg L^−1^. (b) extraction chromatographic analysis of river water sample. (c) 5 μg L^−1^ mixed standard solution. 1. phenanthrene, 2. anthracene, 3. fluoranthene, 4. pyrene, 5. Benzo[a]anthracene, 6. Benzo[b]fluoranthene; Table S1: Specific surface area and pore structure parameters of Fe_3_O_4_, Fe_3_O_4_@SiO_2_ and Fe_3_O_4_@SiO_2_@VAN; Table S2: Physico-chemical characteristics of nine PAHs listed by USEPA, Table S3: Selectivity adsorption parameters of Fe_3_O_4_@SiO_2_@VAN for PAHs.
