# Supplementary material for: Fe3O4@SiO2@VAN Nanoadsorbent Followed by GC-MS for the Determination of Polycyclic Aromatic Hydrocarbons at Ultra-Trace Levels in Environmental Water Samples
Source: Nanomaterials (Basel). 2022 Aug 24;12(17):2921. doi: 10.3390/nano12172921 (PMC9458231; doi:10.3390/nano12172921)
Supplement: Supplementary file 1 [file nanomaterials-12-02921-s001.zip › nanomaterials-1859704-supplementary.pdf]

nanomaterials

MDPI

Supplementary Materials

Fe_3O_4@SiO_2@VAN Nanoadsorbent Followed by GC-MS for the Determination of Polycyclic Aromatic Hydrocarbons at Ultra-Trace Levels in Environmental Water Samples

Yu Tian [1], Zhigang Xu [1,*], Zhimin Liu [1], Xiaoxi Si [2,3,*], Fengmei Zhang [3] and Wei Jiang [3]

1 Faculty of Science, Kunming University of Science and Technology, Kunming 650500, China; yutian_kmust@163.com (Y.T.); lab_chem@126.com (Z.L.)
2 Faculty of Environmental Science & Engineering, Kunming University of Science and Technology, Kunming 650500, China
3 Yunnan Key Laboratory of Tobacco Chemistry, R&D Center of China Tobacco Yunnan Industrial Co., Ltd., Kunming 650231, China; zhangfm2008@126.com (F.Z.); ppp4466@163.com (W.J.)
* Correspondence: xuzgkmust@gmail.com (Z.X.); sixiaoxi2006@126.com (X.S.)

Abbreviations list

No.	Abbreviations	Full name	No	Abbreviations	Full name
1	PAHs	Polycyclic aromatic hydrocarbons	16	PH	phenol
2	PHE	phenanthrene	17	TEOS	tetraethyl orthosilicate
3	AN	anthracene	18	DCM	dichloromethane
4	FA	fluoranthene	19	GC-MS	gas chromatography - mass spectrometry
5	PY	pyrene	20	GC	gas chromatography
6	BaA	benzo[a]anthracene	21	XRD	X-ray diffraction
7	BbFA	benzo[b]fluoranthene	22	SEM	scanning electron microscopy
8	VAN	vancomycin	23	TEM	transmission electron microscope
9	BPA	bisphenol A	24	FT-IR	fourier transform-infrared
10	BPB	bisphenol B	25	BET	Brunauer–Emmett–Teller
11	PEG-2000	polyethylene glycol-2000	26	VSM	vibra sample magnetometer
12	DMF	*N*, *N*-dimethylamide	27	MSPE	magnetic solid-phase extraction
13	HCL	hydrochloric acid	28	SPE	solid-phase extraction
14	EtOH	ethanol	29	ASE	Accelerated solvent extraction
15	Py	pyridine	30	MNPs	magnetic nanoparticles

S1. Characterization

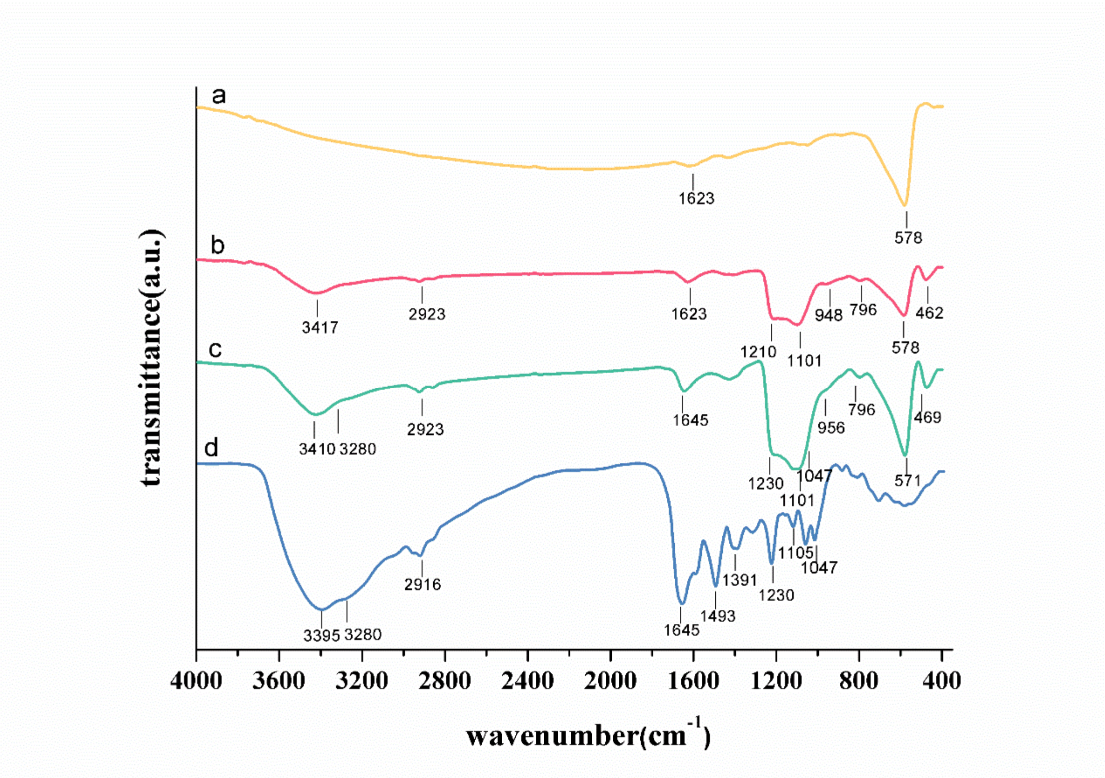

Figure S1. FT-IR spectra of (**a**) Fe_3O_4, (**b**) Fe_3O_4@SiO_2, (**c**) Fe_3O_4@SiO_2@VAN and (**d**) VAN.

Table S1. Specific surface area and pore structure parameters of Fe_3O_4, Fe_3O_4@SiO_2 and Fe_3O_4@SiO_2@VAN.

Sample	Specific Surface Area (m^2/g)	Pore Volume (cc/g)	Average Pore Size (nm)
Fe_3O_4	18.715	0.128	27.349
Fe_3O_4@SiO_2	19.446	0.137	28.100
Fe_3O_4@SiO_2@VAN	22.048	0.108	19.566

S2. Optimization of Extraction Conditions

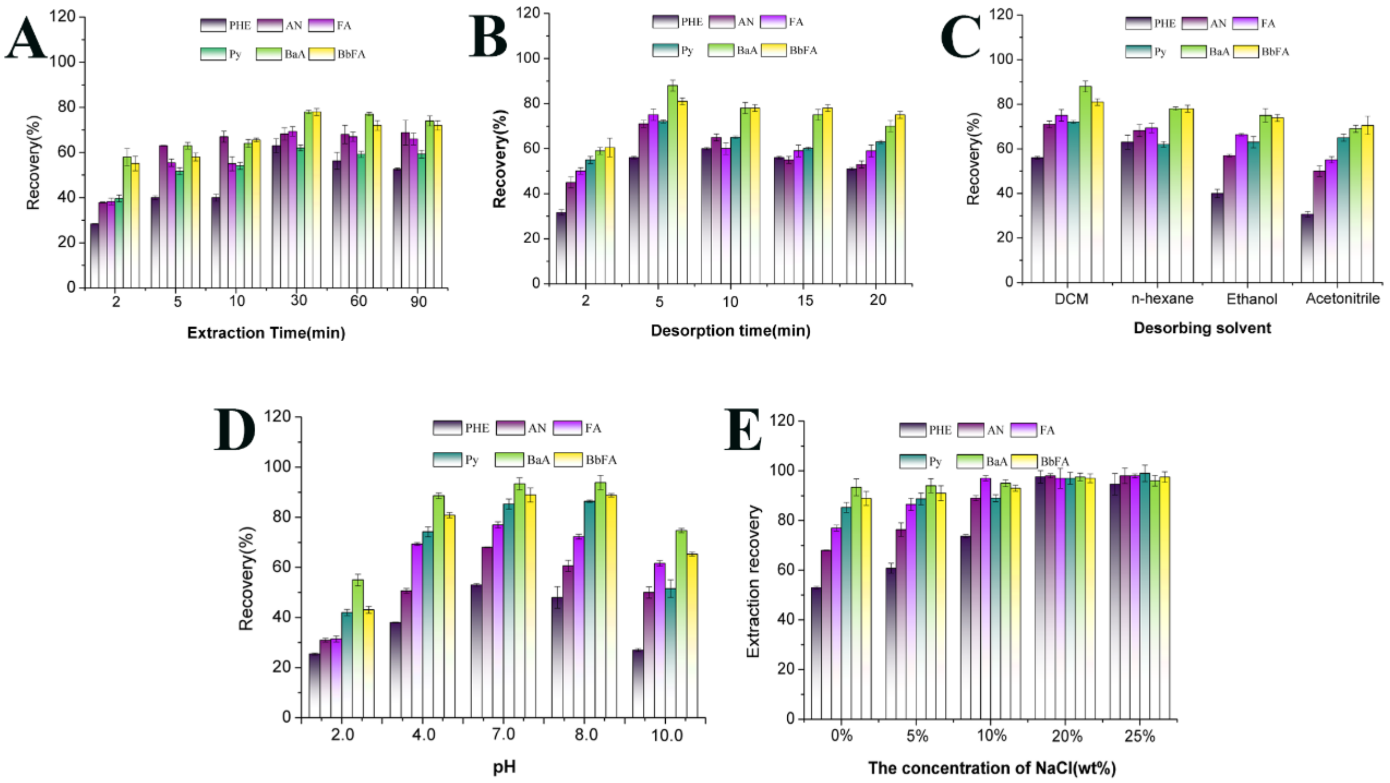

Figure S2. (**A**). Extraction time optimization; (**B**). Desorption time optimization; (**C**). Desorption solvent optimization; (**D**). Solution pH optimization; (**E**). The effect of solution ionic strength on the extraction effect of three kinds of PAHs.

Table S2. Physico-chemical characteristics of nine PAHs listed by USEPA.

Name	Abbreviation	S_w (mg L^{-1})	logK_{ow}
Naphthalene	Nap	31.5	3.37
Acenaphthene	ACN	3.93	3.92
Flurene	Flo	1.69	1.7
Phenanthrene	PHE	1.15	4.57
Anthracene	AN	1.08	4.54
Fluoranthene	FA	0.21	5.22
Pyrene	PY	0.13	5.18
Benz(a)anthracene	BaA	0.009	5.91
Benzo(b)fluoranthene	BbFA	0.002	5.80

Table S3. Selectivity adsorption parameters of $Fe_3O_4@SiO_2@VAN$ for PAHs.

Analytes	Extraction Recovery (%)	*EF*	$SC_{PHE/c}$	$SC_{AN/c}$	$SC_{FA/c}$	$SC_{PY/c}$	$SC_{BaA/c}$	$SC_{BbFA/c}$
PHE	97.0	51.2	/	/	/	/	/	/
AN	98.0	58.0	/	/	/	/	/	/
FA	97.0	53.0	/	/	/	/	/	/
PY	97.0	52.6	/	/	/	/	/	/
BaA	97.5	54.0	/	/	/	/	/	/
BbFA	97.0	50.0	/	/	/	/	/	/
ACN	44.3	11.0	4.7	5.3	4.8	4.8	4.9	4.5
BPA	5.0	2.28	22.5	25.4	23.2	23.1	23.7	21.9
BPB	5.6	1.7	30.1	34.1	31.1	30.1	31.7	29.4
pyridine	0.0	0.0	0.0	0.0	0.0	0.0	0.0	0.0
phenol	0.0	0.0	0.0	0.0	0.0	0.0	0.0	0.0

Abbreviations:
SC_{PHE} is selectivity coefficient of PHE.
$SC_{AN/c}$ is selectivity coefficient of AN.
$SC_{FA/c}$ is selectivity coefficient of FA.
$SC_{PY/c}$ is selectivity coefficient of PY.
$SC_{BaA/c}$ is selectivity coefficient of BaA.
$SC_{BbFA/c}$ is selectivity coefficient of BbFA.

S3. The Chromatogram of Environmental Water Sample

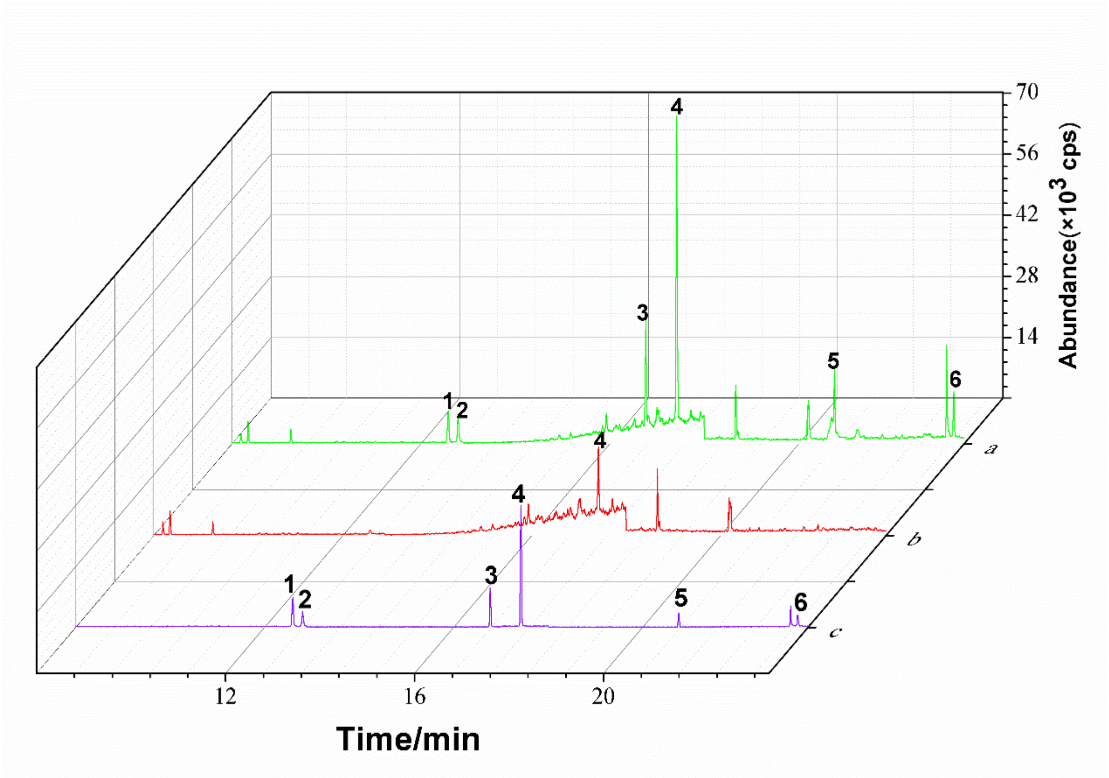

Figure S3. Selected ion chromatogram of Laoyuhe River water sample. a). extraction chromatographic analysis after adding 1 μg/L. b). extraction chromatographic analysis of river water sample. c) 5 μg/L mixed standard solution. 1. phenanthrene, 2. anthracene, 3. fluoranthene, 4. pyrene, 5. Benzo[a]anthracene, 6. Benzo[b]fluoranthene.
